# The Impact of Concurrent Proton Pump Inhibitors on Nivolumab Response in Metastatic Non-Small Cell Lung Cancer: A Multicenter Real-Life Study

**DOI:** 10.3390/medicina62010214

**Published:** 2026-01-20

**Authors:** Engin Hendem, Mehmet Zahid Koçak, Ayşegül Merç Çetinkaya, Gülhan Dinç, Melek Çağlayan, Muzaffer Uğraklı, Dilek Çağlayan, Murat Araz, Melek Karakurt Eryılmaz, Abdullah Sakin, Orhan Önder Eren, Ali Murat Tatlı, Çağlayan Geredeli, Mehmet Artaç

**Affiliations:** 1 Department of Medical Oncology, School of Medicine, Necmettin Erbakan University, Konya 42080, Turkey; mehmetzahidkocak@hotmail.com (M.Z.K.); mugrakli@gmail.com (M.U.); dilekcaglayan88@gmail.com (D.Ç.); zaratarum@yahoo.com (M.A.); drangelkarakurt@hotmail.com (M.K.E.); mehmetartac@yahoo.com (M.A.); 2 Department of Medical Oncology, School of Medicine, Akdeniz University, Antalya 07070, Turkey; aysegulmerc@akdeniz.edu.tr (A.M.Ç.); alimurattat@hotmail.com (A.M.T.); 3 Department of Medical Oncology, Prof. Dr. Cemil Taşcıoğlu City Hospital, İstanbul 34384, Turkey; gulhanduyul@hotmail.com (G.D.); caglayange@hotmail.com (Ç.G.); 4 Department of Medical Oncology, School of Medicine, Selcuk University, Konya 42130, Turkey; melek_caglayan88@hotmail.com (M.Ç.); droneren@hotmail.com (O.Ö.E.); 5 School of Medicine, Medipol University, Bahçelievler, İstanbul 34196, Turkey; drsakin@hotmail.com

**Keywords:** proton pump inhibitors, nivolumab, Carcinoma, non-small-cell lung, progression-free survival, overall survival, drug interactions, microbiota

## Abstract

*Background and Objectives*: Clinically meaningful drug–drug interactions may be overlooked in oncology. Proton pump inhibitors (PPIs) may modulate outcomes with immune checkpoint inhibitors (ICIs) by altering the gut microbiome, altering the immune milieu, and affecting transporter interactions. We evaluated whether concomitant PPI use affects survival among patients with metastatic non-small cell lung cancer (NSCLC) treated with nivolumab. *Materials and Methods*: We retrospectively included patients with metastatic NSCLC who received second-line nivolumab across five oncology centers (January 2020–June 2023). Patients were grouped as concomitant PPI users vs. non-users. Overall survival (OS) and progression-free survival (PFS) were estimated by the Kaplan–Meier method and compared with the log-rank test; multivariable Cox models assessed independent associations. *Results*: A total of 194 patients were screened, of whom 30 were excluded according to predefined criteria. The final analysis included 164 patients—85 PPI users and 79 non-users. Median OS was 26.1 months (95% CI 15.5–36.7) in PPI users and 29.3 months (22.2–36.4) in non-users; this difference was not statistically significant (*p* = 0.54). Median PFS was 6.2 months (3.7–8.6) in PPI users vs. 10.2 months (7.1–13.2) in non-users (*p* = 0.04). In multivariable analysis, absence of concomitant PPI use (No vs. Yes) was independently associated with longer PFS (HR = 0.52, 95% CI 0.24–0.89, *p* = 0.03), whereas PPI use was not associated with OS (HR = 0.96, 95% CI 0.67–1.61, *p* = 0.83). *Conclusions*: Concomitant PPI use during nivolumab therapy was associated with significantly shorter PFS and a numerical reduction in OS in real-world metastatic NSCLC. Where clinically feasible, the need for PPIs should be re-evaluated before and during ICI therapy.

## 1. Introduction

Nivolumab, a human IgG4 monoclonal antibody against programmed death-1 (PD-1), restores antitumor T-cell activity by blocking PD-1 receptor signaling [1]. It has become a standard second-line option for advanced NSCLC [2]. PPIs are widely prescribed in oncology practice, often for dyspepsia, gastritis, or gastroprotection in patients receiving antiplatelet therapy, and their long-term use is common because of a generally perceived safety profile [3]. Experimental studies have suggested that PPIs may influence tumor biology through effects on intratumoral acidity or other microenvironmental changes [4,5]. In clinical practice, PPI exposure has been correlated with modified outcomes for various oral anticancer agents and certain cytotoxic therapies [6,7,8,9].

Different mechanisms have been proposed, including changes in gut microbiota composition, alterations in systemic immune signaling, and effects on cellular transport pathways [9,10,11,12]. PPIs are known to be associated with the gut microbiome, such as higher levels of *Streptococcus*, *Bifidobacterium*, and *Lactobacillus* [13]. Specific microbial changes, including an increased presence of regulatory T-cell–associated species, may adversely affect antitumor immune responses [14]. Hematologic markers indicative of systemic inflammation have been correlated with PPI exposure [15]. Some reports have shown worse ICI-related outcomes in patients using PPIs [16,17], while others have shown no meaningful association [15,18,19].

Data specifically addressing patients with metastatic NSCLC treated with nivolumab are still scarce. In this multicenter retrospective cohort study, we examined whether concomitant PPI use affected survival outcomes in patients receiving second-line nivolumab for metastatic NSCLC.

## 2. Materials and Methods

### 2.1. Study Design and Patients

**Figure 1:**
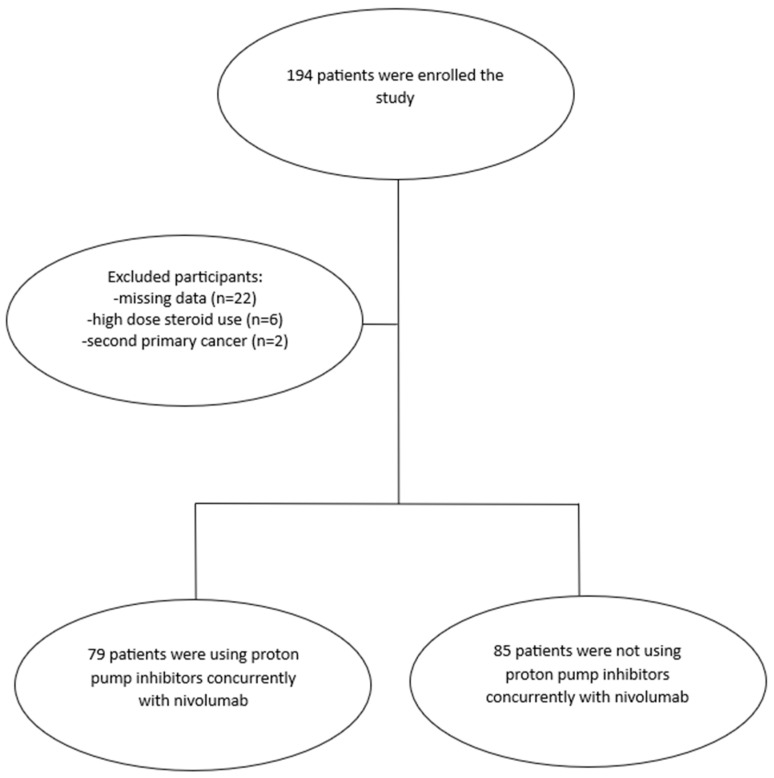
Flowchart of the participants.

This retrospective analysis took place at five medical oncology centers. We assessed patients with metastatic NSCLC who had nivolumab as a second-line therapy from January 2020 to June 2023. In total, 194 patients were screened. Thirty were excluded because clinical information was incomplete, systemic corticosteroid use exceeded 10 mg of prednisone-equivalent for more than two weeks, or a second primary cancer was present. After applying these criteria, 164 patients remained for analysis (85 using PPIs and 79 not using PPIs). The patient selection process is summarized in Figure 1. Ethical approval was obtained from the institutional review board (Approval No. 2023/4609). The median follow-up time was 20.02 months.

### 2.2. Exposure and Outcomes

PPI exposure was recorded at the start of nivolumab treatment. The commonly used PPIs were pantoprazole (40 mg), esomeprazole (40 mg), lansoprazole (30 mg), and rabeprazole (20 mg). Each patient’s indication for PPI therapy (e.g., reflux, gastritis, or prophylaxis) was documented. Patients were grouped as PPI users or non-users.

OS is defined as the duration from initial diagnosis to death from any cause. PFS was assessed from the initial administration of nivolumab until radiographic progression. Objective response and safety outcomes were assessed as secondary endpoints.

### 2.3. Baseline Variables

At the commencement of treatment, multiple baseline characteristics were documented for each patient. These included demographic factors such as age and gender, clinical indicators such as ECOG performance status and body mass index, and recorded comorbidities or smoking history. We also recorded tumor-related information, including PD-L1 expression and whether metastatic spread was limited to a single site or involved multiple sites. Routine laboratory results—particularly hemoglobin and platelet counts—were collected at initiation. Adverse events were evaluated during follow-up in accordance with standard clinical practice.

### 2.4. Statistical Analysis

Categorical variables were compared using the χ^2^ test or Fisher’s exact test, as appropriate. Survival distributions were estimated with the Kaplan–Meier method and compared using the log-rank test. Cox proportional hazards regression models were used to identify predictors of PFS and OS. Statistical analyses were performed using SPSS software (SPSS 20.0; IBM Inc., Chicago, IL, USA). A two-sided *p*-value < 0.05 was considered statistically significant.

## 3. Results

### 3.1. Patient Characteristics

A total of 194 patients with metastatic NSCLC were screened, of whom 164 met the eligibility criteria and were included in the final analysis. Baseline demographic and clinical characteristics of the study population are summarized in Table 1. Among these, 85 patients received a PPI at the initiation of nivolumab therapy, whereas 79 did not. The group’s average age was 63. The distribution of baseline demographic and clinical variables—including sex, ECOG performance status, body mass index, comorbidities, and smoking history—did not differ significantly between PPI users and non-users. Similarly, the proportions of patients with PD-L1 expression in available tumor samples and the number of metastatic sites (single vs. multiple) were comparable across groups. Hemoglobin levels (<10 vs. ≥10 g/dL) and platelet categories (normal range vs. thrombocytosis) also showed no statistically significant variation between the two groups. The frequency of any-grade adverse events attributed to nivolumab was also similar (all *p* > 0.05).

### 3.2. Treatment Response

Among the patients who were receiving PPIs when nivolumab was initiated, one patient (1.2%) achieved a complete response. A partial response was noted in 28 patients (32.9%), and 35 patients (41.2%) maintained stable disease. Progression was observed in 21 individuals (24.7%).

In the cohort without PPI exposure, no complete responses were documented. Partial response occurred in 16 patients (20.3%), stable disease in 37 patients (46.8%), and disease progression in 26 patients (32.9%).

When the overall treatment effect was assessed, the response rates between the two groups were similar (*p* = 0.20). The disease control rate—defined as CR, PR, and SD—was 75.3% in the PPI group and 67.1% in patients not taking PPIs. This difference did not reach statistical significance (*p* = 0.21).

### 3.3. Survival Outcomes

**Figure 2:**
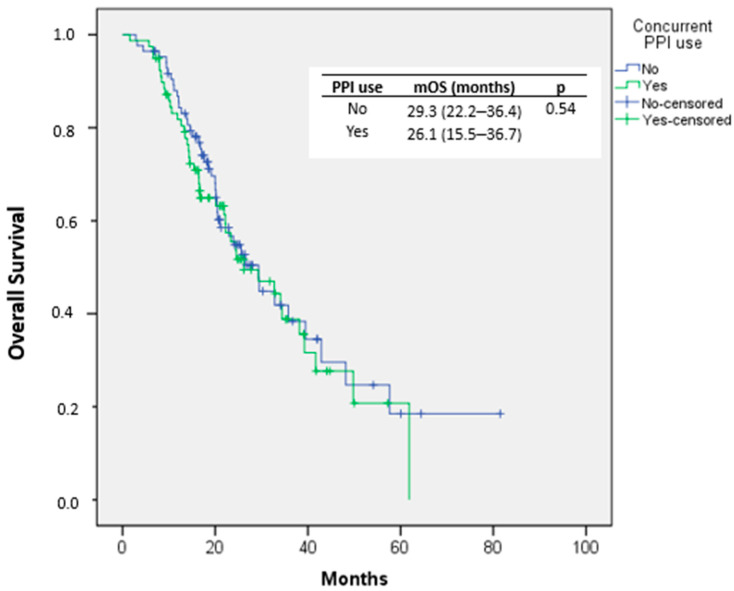
OS curve according to Kaplan–Meier analysis in patients with and without PPI use concomitant with nivolumab.

Median OS was 26.1 months (95% CI, 15.5–36.7) in the PPI group and 29.3 months (95% CI, 22.2–36.4) in the non-PPI group, with no statistically significant difference observed (*p* = 0.54). Survival curves are shown in Figure 2.

**Figure 3:**
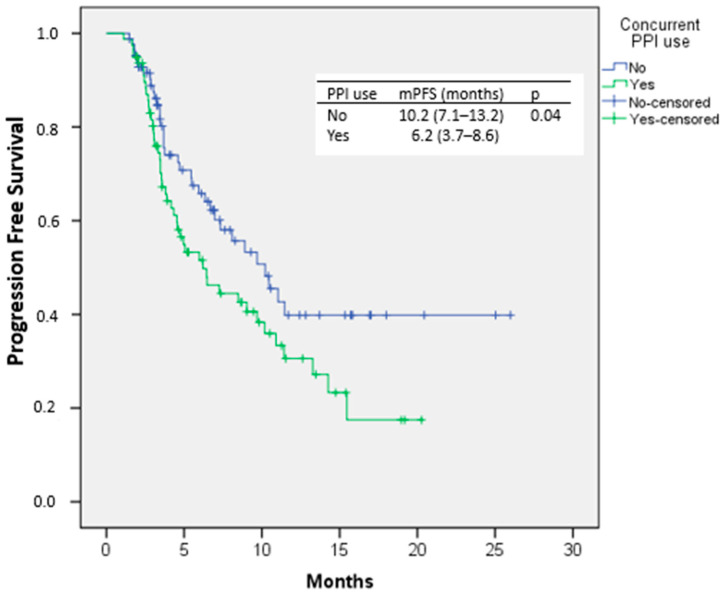
PFS curve according to Kaplan–Meier analysis in patients with and without PPI use concomitant with nivolumab.

Median PFS was significantly shorter among patients receiving PPIs, measuring 6.2 months (95% CI, 3.7–8.6), compared with 10.2 months (95% CI, 7.1–13.2) in non-users (*p* = 0.04). Kaplan–Meier curves for PFS are presented in Figure 3.

### 3.4. Multivariable Analysis

In the multivariable Cox regression model for PFS, three factors remained independently associated with the outcome. Patients younger than 65 years had a lower likelihood of progression compared with older individuals (HR = 0.70, 95% CI 0.38–0.98, *p* = 0.017). Outcomes were also more favorable in patients with a single metastatic site than in those with two or more sites (HR = 0.56, 95% CI 0.35–0.89, *p* = 0.014). Concomitant PPI use remained a negative predictor of PFS. Based on the model coding (No vs. Yes), the reported hazard ratio (HR = 0.52, 95% CI 0.24–0.89, *p* = 0.03) corresponded to an estimated HR of approximately 1.92 for PPI users relative to non-users. Exploratory analyses including C-reactive protein, neutrophil–lymphocyte ratio, and platelet–lymphocyte ratio did not alter the independent association between PPI use and PFS (Table 2).

For OS, age and baseline hemoglobin level were the only variables that retained significance. Survival was better among patients younger than 65 years (HR = 0.78, 95% CI 0.61–0.98, *p* = 0.013). In contrast, individuals with hemoglobin levels below 10 g/dL had a higher mortality risk than those with levels ≥10 g/dL (HR = 1.50, 95% CI 1.11–1.80, *p* = 0.004). Concomitant PPI exposure did not demonstrate an independent association with OS (HR = 0.96, 95% CI 0.67–1.61, *p* = 0.83). Exploratory analyses including C-reactive protein, neutrophil–lymphocyte ratio, and platelet–lymphocyte ratio did not alter the independent association between PPI use and OS (Table 3).

## 4. Discussion

This multicenter retrospective study examined whether concomitant PPI use influences clinical outcomes in patients with metastatic NSCLC receiving second-line nivolumab. In our cohort, PPI exposure at the start of treatment was associated with a significantly shorter PFS and a modest, although statistically nonsignificant, reduction in OS. These associations remained evident after adjustment for major prognostic variables, including age, ECOG performance status, baseline hemoglobin level, and extent of metastatic disease. The observed negative association between PPI use and PFS is in line with accumulating evidence suggesting that commonly prescribed non-oncologic agents can influence ICI efficacy through mechanisms that are not limited to conventional pharmacokinetic interactions [7,9,20,21].

Our findings demonstrate that the gut microbiome mediates immune responses altered by PPI use. PPIs alter intestinal microbial composition by significantly reducing gastric acidity, which fosters the overgrowth of genera such as *Streptococcus*, *Lactobacillus*, *Veillonella*, and *Enterococcus*, and diminishes levels of beneficial commensals like *Akkermansia muciniphila* and *Faecalibacterium prausnitzii* [13]. These microbial shifts are clinically relevant; specific commensals improve antitumor immunity by supporting antigen presentation, dendritic cell function, and CD8+ T-cell priming [14]. In contrast, the abundance of oral and opportunistic bacteria is associated with weakened T-cell activity, increased regulatory T-cells, and reduced interferon gene expression—factors necessary for optimal ICI therapy. Collectively, this mechanistic link provides a biologically plausible reason for reduced nivolumab efficacy in patients taking PPIs, even in the absence of direct hepatic drug interactions.

In addition to the microbiome, various immunologic mechanisms have been suggested. Experimental studies demonstrate that chronic PPI exposure disrupts lysosomal acidification, interferes with antigen processing, and downregulates MHC class I expression, consequently diminishing the capacity of cytotoxic T cells to recognize tumor cells [22,23]. PPIs have been associated with systemic inflammation, as indicated by elevated neutrophil-to-lymphocyte ratios (NLRs), reduced effector-to-suppressor T-cell ratios, and oxidative stress, all of which are independently correlated with impaired responses to ICIs. This pattern is consistent with our clinical findings, particularly the shorter PFS observed among patients receiving PPIs.

Several retrospective studies across tumor types have reported negative associations between PPI use and ICI outcomes [16,17,24,25]. The magnitude of this effect appears to be greatest when PPIs are continued shortly before or during the initial treatment cycles, a pattern consistent with our definition of “concomitant PPI use.” Conversely, some studies—especially post hoc analyses of controlled trials—have not replicated this relationship [15,18,26,27]. Variations in PPI indications, treatment length, microbiome variety among populations, and the presence of co-medications such as antibiotics or corticosteroids likely account for this variability. The sample notably excluded patients with extensive systemic steroid exposure, thereby reducing a significant confounding variable commonly identified in prior studies.

Recent evidence suggests that concomitant PPI use may adversely affect the efficacy of ICIs. Meta-analyses and extensive retrospective studies have demonstrated that PPI exposure during ICI therapy is associated with shorter PFS and OS across multiple cancer types, including NSCLC. These findings support the hypothesis that PPIs may adversely affect immune-mediated tumor control, underscoring the need for the careful evaluation of supportive medications during immunotherapy [28,29,30].

This study provides significant insights into the current literature. First, it focuses specifically on nivolumab in second-line metastatic NSCLC—a setting in which multicenter retrospective cohort data remain limited. Previous studies often combined multiple ICIs or tumor types, making it challenging to isolate drug-specific interactions. Second, the PPI and non-PPI groups exhibited comparable baseline characteristics, suggesting that variations in comorbidities or tumor burden alone are insufficient to account for the study results. Third, although objective response rates were comparable between groups, patients exposed to PPIs progressed more rapidly. Taken together, these findings indicate that the effect of PPIs is more likely related to the durability of immune-mediated tumor control rather than to the initial antitumor response.

The observed association between older age and poorer survival outcomes aligns with immunosenescence, which is characterized by reduced naïve T-cell populations, impaired antigen presentation, and increased suppressor-cell activity [31]. Such age-related immunological alterations may intensify the immunomodulatory effects of PPIs, potentially reducing the effectiveness of ICIs in older patients. In addition, poorer outcomes observed in patients with multiple metastatic sites suggest the presence of a tumor microenvironment that is less favorable for effective immune responses. Tumors with substantial metastatic burden frequently exhibit increased T-cell exhaustion, elevated cytokine-mediated immunosuppression, and greater clonal heterogeneity, all of which contribute to reduced responsiveness to ICIs [32].

In our cohort, PPI use was not associated with a significant difference in OS, despite its clear association with shorter PFS. Crossover to subsequent lines of therapy, such as chemotherapy or targeted agents, may diminish differences in OS. Second, OS is influenced by multiple factors independent of nivolumab efficacy, including performance status decline, intercurrent illness, and treatment tolerability. Third, PFS is often more sensitive than OS to subtle immunologic disruptions, particularly those involving the tumor microenvironment. Our findings echo other real-world and retrospective reports in which PPI use selectively impaired PFS while leaving OS unchanged [17,18]. This further supports the hypothesis that PPIs interfere with sustained, but not initial, ICI-mediated tumor control.

These findings have practical implications for everyday oncology care. PPIs are frequently prescribed to cancer patients and are often continued without reassessment of their indication. In patients who are starting ICIs, particularly nivolumab, concurrent PPI use should therefore be reviewed on an individual basis. In cases where gastric protection is necessary, options such as temporary use, dose reduction, or alternative acid-suppressive strategies may be considered. Considering the extensive utilization of PPIs, restricting unnecessary exposure may have significant clinical implications. Our findings indicate that supportive medications warrant evaluation not only for symptom management but also for their potential influence on the efficacy of immune-mediated treatments.

From a methodological standpoint, several aspects of this study merit attention. It reflects routine clinical practice across multiple centers, which supports the external validity of the findings. The exclusion of patients receiving prolonged systemic corticosteroids helped limit major confounding factors commonly encountered in real-world analyses. In addition, the inclusion of detailed baseline laboratory data and comprehensive information on metastatic burden enabled a more robust evaluation of prognostic factors. Furthermore, nivolumab was uniformly available across all participating centers, which helped reduce variability associated with treatment availability and sequencing between centers.

Alongside its strengths, several limitations warrant consideration. Due to the retrospective design and sample size considerations, propensity score matching was not performed. The retrospective design of the study limits the ability to exclude residual confounding fully. Differences in the clinical indication, dosage, and duration of PPI use may have contributed to exposure heterogeneity that could not be fully addressed in the analysis. In addition, the lack of concurrent biological assessments—such as gut microbiome composition, inflammatory markers, or immune cell profiling—restricts detailed mechanistic interpretation. Although the cohort represents a substantial patient population worldwide receiving second-line therapy for metastatic NSCLC, the sample size may have been insufficient to detect modest differences in OS or to support comprehensive subgroup analyses. Overall, these limitations underscore the importance of future studies that incorporate longitudinal exposure assessment together with translational biomarkers to more clearly define causal relationships. Furthermore, residual confounding factors cannot be ruled out due to the retrospective design and the lack of data regarding variables such as antibiotic exposure and PPI duration.

Retrospective data provide useful signals but are not sufficient to fully define the relationship between PPI exposure and immune-related treatment outcomes. Clarifying this issue will require studies in which patients are followed prospectively, and immune-related changes are recorded over time. In parallel, laboratory-based analyses focusing on gut microbiota composition, inflammatory markers, and T-cell activity could help place the observed clinical findings into a biological context. From a clinical standpoint, it is still unclear whether routine PPI use during ICI is required in all patients or if alternative strategies could adequately control symptoms without affecting patient comfort. In addition, whether discontinuing PPIs before treatment initiation has any meaningful effect on microbiome recovery or clinical outcomes remains an open question.

In this multicenter cohort, patients treated with nivolumab who were exposed to PPIs had shorter PFS, while no clear difference in OS was observed. Clinicians should periodically re-evaluate the indication for chronic PPI therapy during ICI treatment. Further studies are needed to better characterize the interaction between PPI exposure during treatment, host–microbiome interactions, and the immune response.

### Limitations

The retrospective design, potential residual confounding, and heterogeneity in PPI agent and duration limit causal inference. Additionally, PPI exposure was defined only at the baseline, and time-varying changes in PPI use could not be evaluated.

## 5. Conclusions

Concomitant PPI use during nivolumab therapy in metastatic NSCLC was associated with shorter PFS and a numerical OS decrement. Prospective studies are warranted, but clinicians should carefully evaluate the necessity and duration of PPIs in patients receiving ICIs.

## Figures and Tables

**Table 1:** General characteristics of the study population.

Characteristics (*n*)		Concurrent Proton Pump Inhibitor Usage with Nivolumab		
		No	Yes	*p*
Age	<65	54 (63.5%)	45 (57%)	0.39
	≥65	31 (36.5%)	34 (43%)	
Gender	Female	13 (15.3%)	14 (17.7%)	0.67
	Male	72 (84.7%)	65 (82.3%)	
Body Mass Index	<25 kg/m^2^	49 (57.6%)	47 (59.5%)	0.81
	≥25 kg/m^2^	36 (42.4%)	32 (40.5%)	
Comorbidity	No	43 (50.6%)	37 (46.8%)	0.63
	Yes	42 (49.4%)	42 (53.2%)	
Smoking	No	16 (18.8%)	12 (15.2%)	0.53
	Yes	69 (81.2%)	67 (84.8%)	
PDL-1	Unknown	23 (27.1%)	25 (31.6%)	0.81
	<1%	30 (35.3%)	26 (32.9%)	
	≥1%	32 (37.6%)	28 (35.4%)	
ECOG-PS	0	22 (25.9%)	17 (21.5%)	0.7
	1	42 (49.4%)	44 (55.7%)	
	2	21 (24.7%)	18 (22.8%)	
Hemoglobin	<10 g/dL	40 (47.1%)	42 (53.2%)	0.43
	≥10 g/dL	45 (52.9%)	37 (46.8%)	
Platelet	Normal	76 (89.4%)	70 (88.6%)	0.86
	Thrombocytosis	9 (10.6%)	9 (11.4%)	
Metastatic side	1	36 (42.4%)	30 (38%)	0.56
	≥2	49 (57.6%)	49 (62%)	
Any grade adverse event of nivolumab	No	68 (80%)	58 (73.4%)	0.31
	Yes	17 (20%)	21 (26.6%)	
Response rate	Complete	1 (1.2%)	0 (0%)	0.2
	Partial	28 (32.9%)	16 (20.3%)	
	Stable	35 (41.2%)	37 (46.8%)	
	Progression	21 (24.7%)	26 (32.9%)	

**Table 2:** Univariate and multivariate analyses of risk factors associated with PFS.

Progression Free Survival	Univariate Analyses			Multivariate Analyses		
	Hazard Ratio	95% Confidence Interval	*p*	Hazard Ratio	95% Confidence Interval	*p*
Age						
<65 vs. ≥65	0.55	0.35–0.85	0.008	0.70	0.38–0.98	0.017
Gender						
Female vs. Male	1.05	0.58–1.91	0.85	-	-	-
Body mass index						
<25 vs. ≥25 kg/m^2^	1.44	0.91–2.28	0.11	-	-	-
Comorbidity						
No vs. Yes	0.98	0.63–1.51	0.92	-	-	-
Smoking						
No vs. Yes	1.25	0.69–2.27	0.45	-	-	-
PDL-1						
Unknown	Reference		0.13	-	-	-
<1%	0.62	0.37–1.06	0.08	-	-	-
≥1%	0.63	0.37–1.07	0.09	-	-	-
Hemoglobin						
<10 vs. ≥10 g/dL	2.04	1.31–3.19	0.002	1.48	0.93–2.38	0.09
Platelet						
Normal vs. t. cytosis	0.77	038–1.55	0.46	-	-	-
Metastatic side						
1 vs. ≥2	0.62	0.39–0.98	0.04	0.56	0.35–0.89	0.014
C-Reactive protein	1.03	0.99–1.10	0.1	-	-	-
Neutrophil–lymphocyte ratio						
≤4.76 vs. >4.76	0.78	0.44–37	0.39	-	-	-
Platelet–lymphocyte ratio						
≤232.1 vs. >232.1	0.58	0.33–1.02	0.06	-		
Best response						
Complete	Reference		0.1	-	-	-
Partial	0.33	0.04–2.52	0.28	-	-	-
Stable	0.55	0.07–4.06	0.56	-	-	-
Progression	1.23	0.15–9.06	0.83	-	-	-
Concurrent PPI using						
No vs. Yes	0.64	0.41–0.98	0.04	0.52	0.24–0.89	0.03

**Table 3:** Univariate and multivariate analyses of risk factors associated with OS.

Overall Survival	Univariate Analyses			Multivariate Analyses		
	Hazard Ratio	95% Confidence Interval	*p*	Hazard Ratio	95% Confidence Interval	*p*
Age						
<65 vs. ≥65	0.53	0.34–0.81	0.004	0.78	0.13–0.80	0.013
Gender						
Female vs. Male	1.30	0.74–2.28	0.35	-	-	-
Body mass index						
<25 vs. ≥25 kg/m^2^	1.26	0.81–1.95	0.29	-	-	-
Comorbidity						
No vs. Yes	0.72	0.47–1.11	0.14	-	-	-
Smoking						
No vs. Yes	0.83	0.45–1.51	0.54	-	-	-
PDL-1						
Unknown	Reference		0.96	-	-	-
<1%	1.02	0.60–1.73	0.94	-	-	-
≥1%	1.07	0.63–1.81	0.79	-	-	-
Hemoglobin						
<10 vs. ≥10 g/dL	2.39	1.53–3.73	<0.001	1.50	1.11–1.80	0.004
Platelet						
Normal vs. t. cytosis	1.46	0.79–2.69	0.22	-	-	-
Metastatic side						
1 vs. ≥2	0.68	0.43–1.07	0.09	-	-	-
C-Reactive protein	0.99	0.98–1.20	0.28	-	-	-
Neutrophil-lymphocyte ratio						
≤4.76 vs. >4.76	0.79	0.39–1.60	0.52	-	-	-
Platelet-lymphocyte ratio						
≤232.1 vs. >232.1	1.72	0.87–3.37	0.11	-	-	-
Best response						
Complete	Reference		0.001	Reference		0.004
Partial	0.52	0.07–3.99	0.53	0.80	0.21–2.24	0.58
Stable	0.70	0.09–5.18	0.73	0.75	0.30–12.2	0.39
Progression	1.58	0.21–11.67	0.65	4.95	0.59–20.14	0.13
Concurrent PPI using						
No vs. Yes	0.87	0.57–1.34	0.54	0.96	0.67–1.61	0.83

## Data Availability

Available from the corresponding author upon reasonable request.

## Abbreviations

The following abbreviations are used in this manuscript.

PPIs	Proton pump inhibitors
ICIs	İmmune checkpoint inhibitors
NSCLC	Non-small cell lung cancer
OS	Overall survival
PFS	Progression-free survival
PD-1	Programmed death-1
